# Enhancing interoceptive sensibility through exteroceptive–interoceptive sensory substitution

**DOI:** 10.1038/s41598-024-63231-4

**Published:** 2024-06-27

**Authors:** Oran Goral, Iddo Yehoshua Wald, Amber Maimon, Adi Snir, Yulia Golland, Aviva Goral, Amir Amedi

**Affiliations:** 1 Baruch Ivcher Institute for Brain, Cognition, and Technology, Reichman University, Herzliya, Israel; 2 Sagol Center for Brain and Mind, Reichman University, Herzliya, Israel; 3 Digital Media Lab, Bremen University, Bremen, Germany; 4 Computational Psychiatry and Neurotechnology Lab, Ben Gurion University, Be’er Sheva, Israel

**Keywords:** Body awareness, Biofeedback, Human Computer Interaction, Interoception, Respiration, Sensory substitution, Human behaviour, Sensory processing

## Abstract

Exploring a novel approach to mental health technology, this study illuminates the intricate interplay between exteroception (the perception of the external world), and interoception (the perception of the internal world). Drawing on principles of sensory substitution, we investigated how interoceptive signals, particularly respiration, could be conveyed through exteroceptive modalities, namely vision and hearing. To this end, we developed a unique, immersive multisensory environment that translates respiratory signals in real-time into dynamic visual and auditory stimuli. The system was evaluated by employing a battery of various psychological assessments, with the findings indicating a significant increase in participants' interoceptive sensibility and an enhancement of the state of flow, signifying immersive and positive engagement with the experience. Furthermore, a correlation between these two variables emerged, revealing a bidirectional enhancement between the state of flow and interoceptive sensibility. Our research is the first to present a sensory substitution approach for substituting between interoceptive and exteroceptive senses, and specifically as a transformative method for mental health interventions, paving the way for future research.

## Introduction

“The only things we perceive are our perceptions”^1^. Perception consists of exteroception, which refers to the perception of the external world through sight, sound, and so on; and interoception, which refers to the perception of the internal world through visceral senses such as respiration^2^. Combined, these forms of perception provide an interpretation of the world that encompasses us, and that which resides within us.

### Connections between the senses

When it comes to exteroception, an ever-growing body of knowledge acquired in recent years indicates that there are connections between what were classically referred to as individual senses (vision, hearing, etc.). Many of these connections between the senses were surfaced and elucidated through studies pertaining to sensory substitution^3–5^. Sensory substitution is distinct in that it employs a technological system or human–machine interface which serves to transduce sensory information from alternative sensors to the human sensory system, and subsequently relay them to the brain^6^. These studies take sensory perception to the extreme, allowing us to convey sensory information from one sense via another. For example, utilizing an algorithm that translates and conveys visual images via sound, it has been shown that visual areas of the brain can respond to audio soundscapes^3,7,8^. While this has been extensively studied with respect to exteroceptive senses, to our knowledge, it has not been explored with relation to interoceptive senses.

In addition to the surfacing of connections between the senses, there is a burgeoning wealth of information shedding light on the connections between the physical body, and the brain^9–11^. It is possible that similar to the knowledge acquired about the underlying mechanisms of the exteroceptive senses through sensory substitution studies, employing sensory substitution techniques to interoceptive senses could inform about the mechanisms underlying interoception.

### Connections between the body and emotions

Current research indicates that perceiving or experiencing emotion comes about from the combination of sensory and motor information^12^, and there are indications that emotion perception arises from the merging of exteroceptive information with interoceptive information^13^. Moreover, there is now evidence of connections between bodily representations and emotion in the brain. Since the initial discovery of body mappings in the brain by Penfield^14^, numerous additional body maps have been identified^15,16^. Significantly, specific exploration in recent years has indicated that there is an overlap between some of these newly identified homunculi and brain networks associated with emotional processing and nodes of the default mode network. These body representations in nodes of the default mode network are of particular interest due to the importance of the balance between the default mode network and the extrinsic network for mental wellness, with over-activity of the default mode network being correlated with numerous psychological and psychiatric conditions such as anxiety and schizophrenia^17^.

One example of a particular area of overlap is the precuneus^15^, known to be correlated with body awareness, among its numerous roles. Particularly body awareness with relation to the sensory systems and the integration of information from them. Damage to the precuneus has been shown to underlie body awareness disorders^18^, and activation of the precuneus has been linked to interoceptive information processing^19^. The body representations identified in the precuneus^15^, taken together with recent research associating a particular area of the precuneus with the formation of one’s bodily self and body schema^20^, further solidify the link between the brain’s perception of the physical body and emotions.

### Exteroceptive–interoceptive sensory substitution

Following the aforementioned, we wish to explore how one can use a sensory substitution inspired system and method for conveying interoceptive information through exteroceptive processes. Furthermore, we propose integrating the principles of sensory substitution with knowledge about body representations in emotion processing areas of the brain (particularly areas correlated with the body and interoception), as means for supporting emotional regulation. In this manuscript, we propose an initial implementation of such a system which applies these ideas. The proposed implementation utilizes respiration as the interoceptive sense, and a visual and auditory representation of one’s breath as the exteroceptive stimulus. In this manner the proposed system utilizes a phenomenon often discussed with respect to sensory substitution, that of distal attribution. Distal attribution is forming an intimate connection between an internal signal, and an external one^21,22^. In this context, the proposed system is primed to aid the user in perceiving interoceptive respiration signals, through exteroceptive visualization and sound both responsive to the breathing patterns in real time, also known as embreathment^23^.

This is particularly interesting, as interoception is strongly related to wellbeing, so such an approach could potentially be utilized for designing sensory stimuli that affects wellbeing. As a key factor in human experience, interoception plays a significant role in different psychological processes, in particular cognitive and emotional ones. It was previously linked to emotional regulation, behavioral regulation, and decision making^2,13,24–27^. Furthermore, it is widely assumed that this perception of the body has a strong relation to relaxation and well-being^27^. Interoception is often discussed as a core element of mindfulness, and as an effective mechanism behind it^27^. Popular types of mindfulness practices, like breathing meditation or body scan, guide practitioners to redirect their attention to their bodies. These types of mindfulness practices were associated with different benefits: improved stress management, emotional regulation, and the ability to understand and cognitively analyze the state of one’s body^27–30^.

We chose breathing as the interoceptive signal for the system, since it is one of the only interoceptive signals that can be controlled voluntarily at will^31^. Due to this unique characteristic of breathing compared to other physiological signals, it has been the basis for many techniques with different goals^32^. Multiple studies, that researched different breathing techniques, reported significant effects on the participants’ relaxation, arousal level, comfort, pleasantness, and attention (for a review see Zaccaro et al.^32^). In traditional meditation practices, breathing is used to induce the practitioner’s relaxation, while reducing their reactivity, sharpening their focus, and improving their general well-being. Modern psychological research supported breathing as an effective tool in all of those fields^32^. These findings opened the door for new studies in additional, sometimes overlapping fields. Practicing regulated breathing techniques has been linked with reduced anxiety, depression, and anger; and improved mood, emotional regulation, and cognitive skills, like memory^32,33^. In neuroimaging studies, breathing practices were found to modulate brain activity, with increased activity in the prefrontal cortex, motor, and parietal cortex, pons, thalamus, and hypothalamus^32^. In general, respiration patterns have been shown to influence different physiological measurements, for example decreasing sympathetic activation, raising heart rate variability, and leading to modifications of the central nervous system^32,34^. The multi-directional connection between the respiration pattern, mental and physiological states has led to the wide use of breathing techniques in biofeedback^35–37^.

Various applications utilize breathing detection to create a meditative effect^38–42^. Most of these applications are based on biofeedback, with the aim of employing external cues to guide the users to control their physiological functions. In this study we employ a similar methodology, albeit through a different approach and with different aims. We built a novel system meant to embody its users’ internal bodily signals, representing an extension, or exportation of their bodily signals outward. The system was meant to link the internal attention of the users with external stimuli that represents their breathing, only to direct this attention back inside, to their internal bodily awareness. We hypothesized that users who experienced embodiment using the system would exhibit higher interoceptive sensibility, and sense of flow and decreased mind wandering all of which indicate immersiveness and positive engagement in the experience. Interoceptive sensibility is a measure defined, according to the accepted model of interoception^24^, as the tendency to be internally focused, and “captures self-reported beliefs about body sensations, which are typically assessed via self-report measures such as questionnaires.”^43^ The two parameters of interoceptive sensibility and flow taken together would indicate the successful substitution between the exteroceptive visual signals and the interoceptive respiration signals, and second, indicate the system could be aimed towards positively influencing one’s state of wellbeing.

## Design and implementation

**Figure 1:**
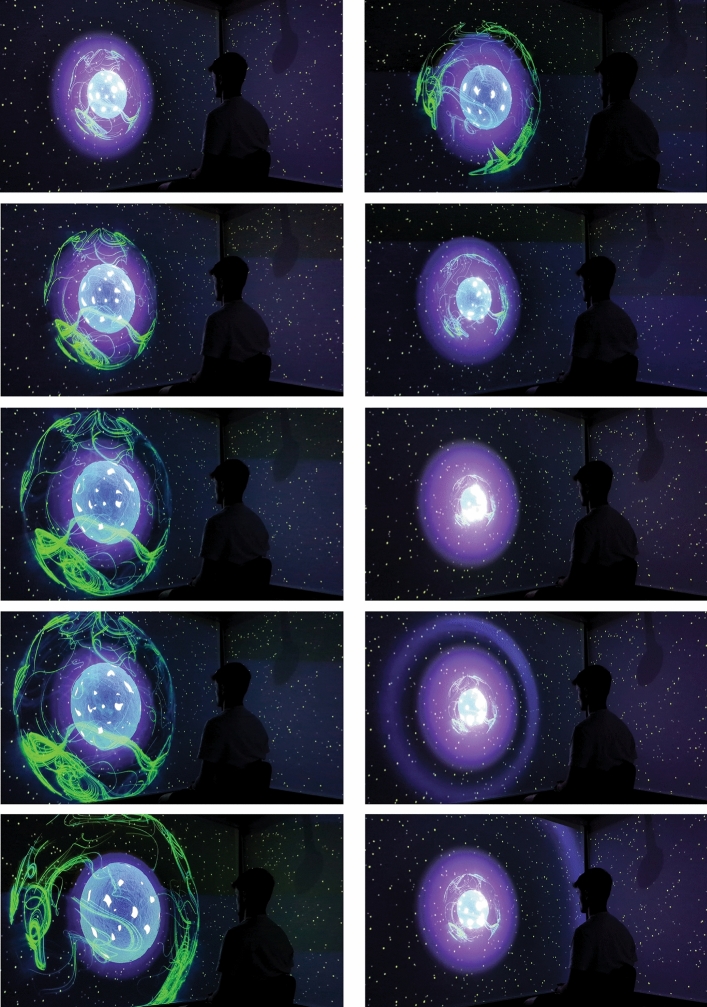
A time lapse of the experience in the room. From top to bottom, inhalation (left) and exhalation (right), including a wave effect at the end of exhalation.

We designed a visual and auditory interaction responsive to the user's breath (Fig. 1). At the center of the experience is a multi-layered sphere. The interaction took place in a unique room installed with a spatial audio system (ambisonics), allowing to simulate auditory spaces, or localizing sounds in space in very high accuracy (12th order). The video environment was projected over three screens surrounding the user, with the majority of the visual interaction taking place in the central screen.

### Design elements and choices

**Figure 2:**
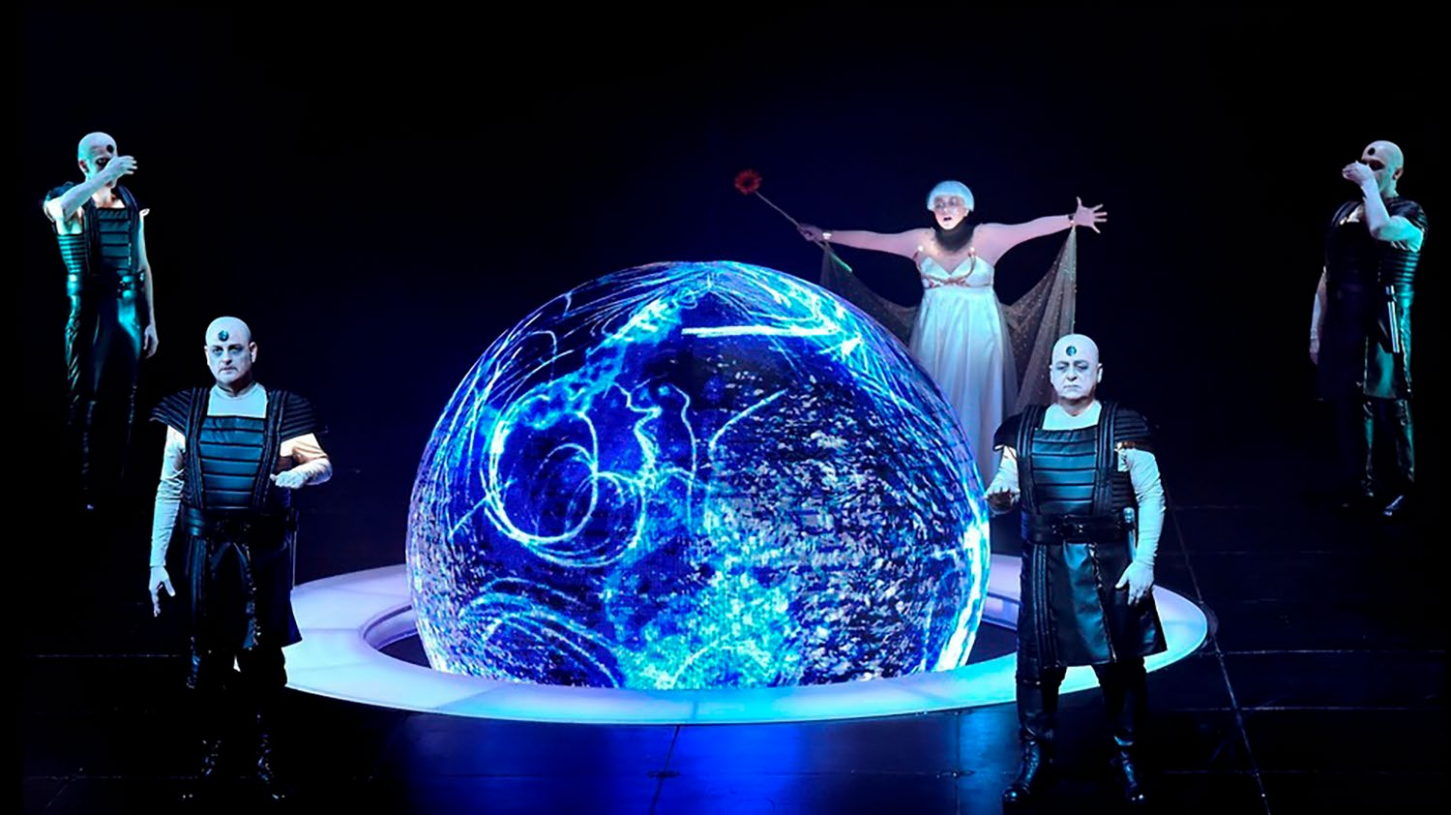
A spherical LED display animation designed by Yoav Cohen, from Salome, The Israeli Opera 2019^45^. Reproduced with permission. Photograph taken by Yossi Zwecker.

Considering the experience as a fully immersive wellbeing oriented experience, we chose to collaborate with an experienced visual artist, drawing inspiration from his previous work (Fig. 2). The system was designed first and foremost to represent the user’s breathing in real-time, and as such almost all of the elements of the environment were in some way responsive to the user’s breath. We also defined the guidelines for the environment to be calm, safe, and positive, with the elements being ambiguous, inviting the user’s subjective interpretation, applying a minimal and abstract design, reminiscent of work such as Char Davies’ Osmose^44^.

The visual scene consisted of a black space populated by glowing, moving particles. In the center of this space, a multi-layered sphere was situated, consisting of three elements—a perforated blue orb; a surrounding, semi-transparent, green sphere; and an inner glowing element. The orb’s behavior was directly mapped to the user’s breath—expanding on inhalation and contracting on exhalation. This direct mapping, simulating the user’s chest or lungs, was meant to create a strong sense of embodiment. The surrounding sphere followed the orb’s movement with a slight delay, enriching the movement with a more organic nature. The glowing element at the heart of the orb, responded with the opposite correlation—expanding on exhalation and contracting on inhalation. This element was designed to encourage extended exhalation, commonly correlated with psychological benefits such as relaxation and positive energy^46,47^. Beyond these basic interaction mechanisms, to further “reward” extended exhalation, we installed a mechanism that triggered a wave of light emitted from the center of the orb whenever the user extended their exhalation to an outstanding period (Fig. 1, right).

The audio component of the experience utilized the spatial audio system installed in the room, and complemented the visual interaction with two elements: (1) an ambient sound composed of multiple frequencies located in different places in space, that slowly circulated back and forth around the participant. (2) a filtered white noise, emanating from the same place as the orb, designed to emit an ocean-wave-like sound upon expansion. All sound distances were set to expand and contract in synchrony to the person’s breathing cycle within the ambisonic environment.

### Technical implementation

**Figure 3:**
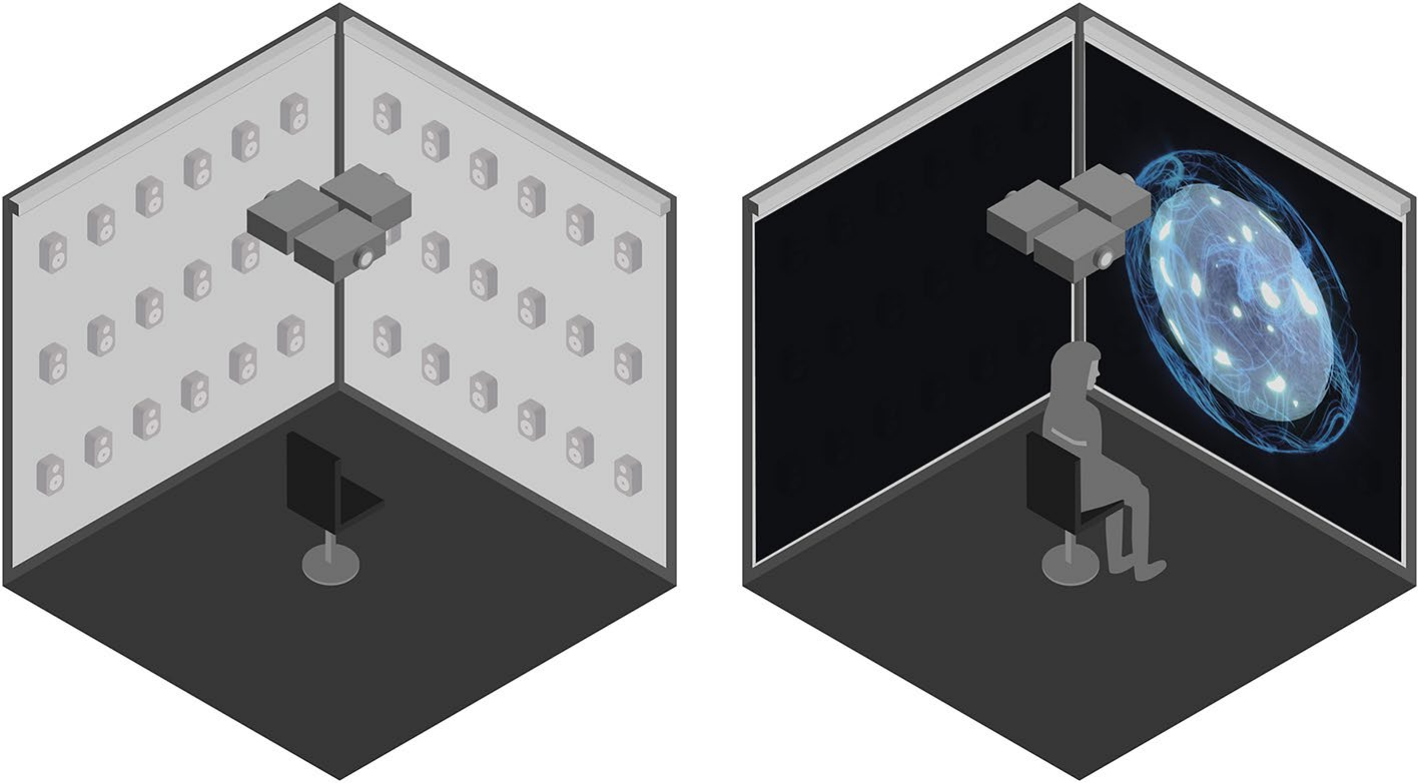
The technical layout of the experiment room (left) and the configuration of the room during the experiment (right).

The experience took place in a cube-shaped, acoustically shielded room (4 × 4 × 4 m), designed to allow for highly controlled 3D experiences. The room is equipped with 97-speakers spread across the ceiling and the walls. In addition, the room is equipped with four high-end Barco projectors and sound-transparent screens surrounding the users’ field of view (Fig. 3). The experience was operated from a separate control room which allowed for an isolated individual experience.

For sensing the user’s breath, a Plux respiration belt (PZT) was used, connected via Bluetooth, and processed by the OpenSignals software. Samples were then sent in real-time via Lab Streaming Layer (LSL) protocol to a Python script for calibration, mapping values to a 0.2–0.8 range according to the averages of the peak values collected throughout the experience in 10 s windows, with the rate of change in mapping parameters limited to 0.01/ms to prevent sudden changes. The resulting signal was then streamed to Max/MSP, where it was smoothed using running average combined with linear interpolation, which were chosen for stability and responsiveness during real-time processing. The processed signal was then sent out to Unity for controlling the visual experience. User Datagram Protocol (UDP) was used to communicate among the various softwares on the network. Within Unity, 0–1 values were mapped to the orb’s size, and the light wave mechanism was triggered whenever a value below 0.3 was hit during exhalation (the value was chosen based on experimentation with the system). Audio was also synthesized in Max/MSP. The breathing 3D locations were encoded via the Spat5 ~ Max/MSP library, and decoded using the AllRad method with 12th order ambisonics.

## Results

Prior to participation in the study, participants were asked to fill the following preliminary trait questionnaires: Multidimensional Assessment of Interoceptive Awareness (MAIA)^48^, Mind-Wandering Questionnaire (MWQ)^49,50^, and State-Trait Anxiety Inventory (STAI-trait)^51^. No significant differences were found between the two study groups in these preliminary measures. During the experiment, participants were asked to fill out state questionnaires post-baseline (following a 3 min baseline session), and post-condition (following the intervention). No significant differences were found between the two study groups in pre-intervention measures. With the exception of the Amsterdam Resting State Questionnaire (ARSQ)^52,53^, all post intervention measure means were significantly higher among participants in the intervention group compared with participants in the control group (Table 1). Bayesian analysis results support those of the frequentist analyses with BF values ranging between 2 and 250 for all comparisons but ARSQ.

**Table 1:** Post intervention measures by study group.

Measure	Control (n = 30)		Intervention (n = 25)		T	P	95% CI	BF*
	Mean	SD	Mean	SD				
Interoceptive sensibility	3.39	0.93	4.27	0.61	− 4.16	< 0.001	− ꝏ– − 0.51	250.15
AFSS	3.19	0.60	3.66	0.47	− 3.12	0.001	− ꝏ– − 0.21	25.41
ARSQ	2.73	0.56	2.86	0.48	–	–	–	0.61
Effort	21.93	19.44	33.33	26.16	− 1.84	0.040	− ꝏ– − 1.01	2.07

*The alternative hypothesis specifies that the control group location is smaller than the intervention group location.

Table 2 presents Pearsons’s r and BF_10_ values for the correlations between post intervention measures. A significant, positive correlation was found between interoceptive sensibility and flow (r = 0.59, p < 0.001). Observed BF_10_ of about 9015 imply a strong correlation between the two variables (i.e., the data are over 9000 times more likely under the alternative hypothesis compared with the null hypothesis).

**Table 2:** Correlations between post intervention measures.

Measure	Interoceptive sensibility	AFSS	Effort	ARSQ
Interoceptive sensibility BF_10_	–			
AFSS BF_10_	0.589** 9015.39	–		
Effort BF_10_	0.040 0.176	− 0.153 0.308	–	
ARSQ BF_10_	0.205 0.504	− 0.044 0.177	0.170 0.355	–

**Correlation significant at p < 0.001.

Aside from the background characteristics included in the first step of the regression model, the final regression model included the following: MAIA mean score, STAI trait score, pre-intervention STAI anxiety score and system use (Table 3). These were found to be significant body-awareness predictors, however, while system use, greater MAIA mean score and STAI trait score predicted greater body-awareness scores, STAI-state anxiety score predicted lower body awareness scores. System use accounted for a significant 10% increase in the variance of body awareness. Overall, the final multivariate regression model explained 65% of the variance in body awareness [F(7, 49) = 13.11, p < 0.001].

**Table 3:** Interoceptive sensibility predictors, results of the multivariate regression analysis.

Predictor	Interoceptive sensibility							
	B	SE	β	Sig	95% CI	BF mean	BF_inc_	R^2^
Constant	0.29	0.75				3.80	1.00	
Step 1								
ADD	− 0.15	0.19	− 0.07	0.43	− 0.53–0.23	− 0.06	0.41	0.13
Meditation	0.11	0.27	− 0.04	0.68	− 0.43–0.65	− 0.02	0.25	
Gender	0.16	0.18	0.08	0.38	− 0.21–0.53	0.07	0.48	
Steps 2–4								
MAIA mean score	0.96	0.19	0.67	0.00	0.58–1.35	0.92	16,803.20	0.55
STAI-trait	0.04	0.01	0.46	0.00	0.02–0.06	0.04	69.45	
STAI-state*	− 0.05	0.01	− 0.43	0.00	− 0.07– − 0.03	− 0.05	249.08	
Step 5								
Condition	0.72	0.17	0.40	0.00	0.39–1.05	0.64	225.07	0.65

Results of the final adjusted regression model.

*Pre-intervention.

Results of the Bayesian linear regression show strong evidence for including condition, MAIA mean score and both STAI-trait scores in the model (adjusted for gender, ADD and meditation). BF_inc_ for these predictors range between 69 and over 16,803 providing further support for the frequentist regression results.

Users of the system scored higher on flow. The final stepwise regression model comprised the following AFSS predictors: MWQ mean score, current STAI anxiety score, current arousal level, system use and the interaction between arousal level and system use (Table 4). MWQ mean scores predicted lower AFSS mean scores while system use predicted greater AFSS scores. In addition, system use accounted for a significant 4% increase in the variance of AFSS, beyond the moderating effect of current arousal as implied by the significant interaction between system use and arousal level. Overall, the final multivariate regression model explained 45% of the variance in AFSS [F(8, 45) = 4.58, p < 0.001].

**Table 4:** Flow (AFSS) predictors, results of the multivariate regression analysis.

Predictor	Flow (AFSS)							
	B	SE	β	Sig	95% CI	BF mean	BF_inc_	R^2^
Constant	3.69	0.58				3.41	1.00	
Step 1								
ADD	− 0.08	0.16	− 0.06	0.63	− 0.40–0.24	− 0.002	0.39	0.08
Meditation	0.29	0.18	0.18	0.11	− 0.07–0.65	0.11	0.94	
Gender	0.36	0.16	0.22	0.02	0.05–0.65	0.20	2.55	
Steps 2–4								
Arousal*	0.001	0.004	0.04	0.78	− 0.007–0.009	− 5.96 × 10^5^	0.43	0.34
MWQ mean score	− 0.21	0.07	− 0.34	0.005	− 0.35–0.07	− 0.17	12.19	
STAI-state*	− 0.02	0.008	− 0.20	0.07	− 0.03–0.001	− 0.007	1.24	
Step 5								
Condition	1.58	0.43	1.34	0.001	0.72–2.45	1.12	72.22	0.38
Step 6								
Arousal by condition interaction	− 0.02	0.006	− 0.96	0.01	− 0.03– − 0.004	− 0.01	7.28	0.45

Results of the final adjusted regression model.

*Pre-intervention.

Further support for the frequentist regression results were also found with the Bayesian linear regression for flow. BF_inc_ values are higher than one for all variables found significant in the frequentist regression model with the highest BF_inc_ observed for condition (BF_inc_ = 72.22) implying that condition is over 77 times higher than the null model in predicting flow.

To better understand the interaction between system use and arousal level, the association between AFSS and current level of arousal was assessed separately for each study group. Results show that AFSS score significantly decreased with increasing arousal level among experimental group participants (b =  − 0.02, p = 0.01, 95% CI − 0.019– − 0.002) but not among control group participants for whom AFSS scores did not change with increasing arousal level.

Mind wandering was differentially affected by initial state. ARSQ predictors included in the final regression model are presented in Table 5. Self-reported meditation experience (compared with low or no experience) predicted lower ARSQ mean scores. Pre-intervention ARSQ scores and PANAS Positive mean scores predicted greater ARSQ scores. The interaction between system use and arousal level was marginally significant (p = 0.055), nevertheless, its addition to the model accounted for a significant 5% increase in the variance of ARSQ. The final multivariate regression model explained 44% in the variance of ARSQ [F(8, 45) = 4.58, p < 0.001].

**Table 5:** Mind wandering (ARSQ) predictors, results of the multivariate regression analysis.

Predictor	Mind wandering (ARSQ)							
	B	SE	β	Sig	95% CI	BF mean	BF_inc_	R^2^
Constant	1.10	0.58				2.90	1.00	
Step 1								
ADD	− 0.22	0.18	− 0.15	0.23	− 0.58–0.14	− 0.05	0.58	0.27
Meditation	− 0.43	0.21	0.24	0.05	− 0.86– − 0.002	− 0.21	1.56	
Gender	0.29	0.18	0.19	0.11	− 0.07–0.64	0.12	1.01	
ARSQ delta*	0.53	0.15	0.43	0.00	0.24–0.82	0.43	40.26	
Steps 2–3								
PANAS Positive	0.35	0.13	0.37	0.01	0.09–0.60	0.15	2.16	0.38
Arousal**	− 0.01	0.01	−0.15	0.35	− 0.02–0.01	0.001	1.04	
Step 4								
Condition	0.75	0.48	0.59	0.12	− 0.22–1.72	0.22	1.00	0.39
Step 5								
Arousal by condition interaction	− 0.02	0.007	− 0.75	0.055	− 0.03–0.00	− 0.026	1.73	0.44

Results of the final adjusted regression model.

*Pre-intervention, **post minus baseline.

Results of the Bayesian linear regression for mind wandering show BF_inc_ > 1 for those predictors that were found significant in the classical regression analysis. As can be seen in Table 5, condition is almost four times as high as the null model in predicting mind wandering.

The association between post ARSQ total score and current level of arousal was assessed separately for each study group. Post intervention ARSQ total scores decreased with increasing arousal level among experimental group participants (b =  − 0.02, p = 0.05, 95% CI − 0.03–0.00) but not among control group participants. This too was supported by the results of the Bayesian analysis (BF_mean_ =  − 0.01, BF_inclusion_ = 1.99 for the experimental group and BF_mean_ =  − 3.591 × 10^−4^, BF_inclusion_ = 0.46 for the control group).

System use was not associated with anxiety or mood. While a significant time effect was found between baseline (mean = 34.10, SD = 7.44) and post-intervention (mean = 31.69, SD = 8.08) STAI mean scores [F(1, 53) = 7.23, p = 0.01; BF_10_ = 5.29], no significant group or interaction effects were found. Similarly, a significant time effect was found for PANAS positive scores (baseline and post-intervention means were 3.38 and 2.85 respectively; F(1, 53) = 21.26, p < 0.001; BF_10_ = 1096.55) no significant group or interaction effects were found. No significant group, time or interaction effects were seen for PANAS negative scores.

## Discussion

In the present study, we investigated and implemented a novel proof of concept inspired by research into sensory substitution, between the exteroceptive senses (vision and hearing) and interoceptive (respiration). To this aim, we designed and implemented a multisensory interactive environment designed to be highly immersive, and represents the user’s respiration. We employed a battery of tests to explore the user’s interoceptive sensibility, flow and mind wandering, among other parameters, and found that the system significantly affected the measures of interoceptive sensibility and flow. These findings, taken together with the high correlation between flow and interoceptive sensibility, support the notion that participants’ attention was directed back to their internal sensations as a result of the external stimuli. This indicates a bidirectional connection between flow state and interoceptive sensibility such that there is a strong relationship between the engagement with the immersive external representation of respiration—and the extent to which the subject is aware of their body signals and sensations. We suggest that this method facilitates one’s ability to be in a state of flow with respect to their internal body sensations.

The findings point to the possible use cases of the system for neuro-wellness, and other potential psychological gains to be explored in future research. This is based on the well established connection between interoception and wellbeing^54,55^ and the known significance of flow to the field of positive psychology^56,57^.

Another interesting finding in the current study was that participants' experience with meditation predicted lower tendency to mind wandering during the experiment condition. This supports previous findings according to which mindfulness meditation is related to reduced mind wandering^49^.

The results of the study showed that users experienced coupling between the multisensory visual and auditory stimuli to their respiratory patterns and responses. We suggest this to be an indication of the system's ability to act as a sensory substitution system, viewing it through the lens of distal attribution. Distal attribution is attributing the cause of a certain sensation to the 3D space externally surrounding one's body. This term more broadly describes the nature of the link between a bodily sensation to the perception of an external object that brings about this sensation^22^. It is commonly investigated with respect to sensory substitution and the perceptual experience with such systems. Prior research on users of sensory substitution systems indicates that when there is a correspondence between movement and sensory stimulation, the users of the system tend to perceive a coupling between the movement and sensory stimulation^21^, which is consistent with the observations from our experiment. As such, this system could provide a basis for further exploration of sensory substitution, and the link between our perceived perceptions and their relationship with information from the external and internal environments. It should be noted that sample size may be viewed as a limitation of the present study. Future research conducted with this system should formally ensure statistical power while determining the sample size in order to corroborate, further explore, or extend upon, the findings of the current findings.

One of the challenges we faced in exploring the effect of our system on one's state interoceptive sensibility, was finding an evaluation method fitting for examining the particular nuances of one’s relevant interoceptive states. As such, we composed a 15-item questionnaire to measure state interoceptive sensibility, the Short State Interoceptive Sensibility questionnaire (SSIS). A similar approach was taken by Blum et al.^58^ with respect to variables for which validated questionnaires are lacking. While this is a limitation of the study, the formulation of this questionnaire is supported by current research that calls for more assessments of interoception, particularly highlighting the significance of doing so while taking into consideration the link between interoceptive and exteroceptive processes^59^. The questions were inspired by the MAIA’s different subscales: noticing, non-distracting, attention regulation, emotional awareness and body listening. The MAIA is a validated and widely used measure for assessing interoceptive awareness^48^. Going forward, we plan to validate the SSIS questionnaire as a measure of state interoceptive sensibility.

We aim to inquire into the neuropsychological mechanisms underlying such exteroceptive–interoceptive sensory substitution by running the study using fMRI. For this purpose, we have developed a system for recreating the real time representation of one’s breath which is MRI compatible. Through such a study we could further explore the possibilities of modulating the balance between default and extrinsic mode networks, a balance that is suggested to be crucial for mental health and wellbeing^17^.

In conclusion, this study explored more than a system, but also a novel principle that could be implemented in different future systems with various aims. Be it for wellness applications, supporting enhanced interoception and flow, to exploring the limits of our sensory perception not only with respect to how we perceive the external world, but also with respect to our internal perceptions.

## Methods

### Participants

Sixty participants were initially recruited for the study, of these five were excluded due to technical device failure during the baseline session. The final sample consisted of 55 participants, mean age was 26.1 (standard deviation, SD = 2.3 years), about 75% (N = 41) females. All participants were recruited through the Reichman University SONA credit system, or volunteered to participate and written informed consent was obtained from each participant. All participants had normal or corrected to normal vision and reported having no known hearing or balance impairments, nor any neurological conditions. Study participants were randomly divided in two groups: 30 (54.5%) in the control group and 25 (45.5%) in the intervention group (i.e., system use). No significant differences were found in background characteristics between the two groups. This study received full ethics approval from the Reichman University Institutional Review Board (IRB) and performed in accordance with the Declaration of Helsinki and all other relevant guidelines and regulations.

### Study procedure

Prior to participation in the study, participants were asked to fill out a number of preliminary trait questionnaires at their own time. These included the Multidimensional Assessment of Interoceptive Awareness (MAIA)^48^, Mind-Wandering Questionnaire (MWQ)^49,50^ State-Trait Anxiety Inventory (STAI-trait)^51^ as well as a demographic questionnaire (further discussed below). Upon entering the laboratory, participants were taken to the experimental room and instructed to take a seat on a chair placed at the center of the room. Following a short explanation regarding the experiment duration and number of phases, the breathing belt was provided and fitted, and they were asked to sit quietly for a three-minute baseline period. During this period no stimulation was given. Following the baseline period, participants remained in the room and were given post-baseline questionnaires, including PANAS^60^, STAI-state, ARSQ^53^, as well as two single items measuring arousal and valence. After completing the questionnaires, participants were informed about the upcoming experimental phase, and were instructed to breathe naturally. Each participant was engaged in an 8 min condition based on their respective group (intervention or control). In the intervention experimental group, the visual and auditory components were fully responsive to the user’s respiration pattern as detailed in the “Design and implementation*”* section. While for the control group, the environment was not responsive to the participants’ breath, rather the visual sphere was displayed as unchanging in size and merely slowly rotating. Upon completion of the breathing experience, participants were asked to complete once again the same set of questionnaires as following baseline, along with the Activity Flow State Scale (AFSS)^61^, SSIS, embodiment questionnaire, and a Single Ease Question (SEQ)^62^ assessing their perceived level of effort (i.e., "Overall, how difficult or easy was it to perform this task") on a 1–100 scale.

### Scales

Participants were asked to fill out a preliminary questionnaire prior to arriving at the lab. Preliminary data collected included participants’ background information (age, sex, education level), ADD diagnosis (yes/no), and level of experience with meditation (responses were rated on a five-point Likert scale ranging from 1 = no experience to 5 = much experience). The preliminary questionnaire also included a range of scales used to measure different aspects of the participant’s well-being, interoceptive sensibility, body awareness and level of anxiety. These included:

- The Multidimensional Assessment of Interoceptive Awareness (MAIA) scale is a 32-item multidimensional self-report measure of interoceptive body awareness. MAIA items were assessed with regards to the occurrence and severity during the previous month, responses were rated on a six-point Likert scale ranging from 1 = never to 5 = always (higher scores represent higher interoceptive awareness). A total MAIA score was calculated as the mean of all 32 items (1–5). The MAIA scale has good psychometric properties in its original form. In the current study, Cronbach’s alpha was 0.914.
- The State-Trait Anxiety Inventory (STAI) scale was used to measure trait stress. STAI responses were rated on a five-point Likert scale ranging from 1 = almost never. to 4 = almost always. A total STAI score was calculated as the sum of all scale items. The STAI scale has good psychometric properties in its original form. In the current study, Cronbach’s alpha was 0.893.
- The Mind-Wandering Questionnaire (MWQ) consists of five items assessing participants’ trait tendency to mind wandering. Items were designated along a six-point Likert scale (1-almost never, 2-very infrequently; 3-somewhat infrequently; 4-somewhat frequently; 5-very frequently; 6-almost always). A total MWQ score was calculated as the mean of all items. The MWQ scale has good psychometric properties in its original form. In the current study, Cronbach’s alpha was 0.847.

Post-baseline and post-condition data included measures of participants' current level of arousal and valence (Rate your level of arousal/comfort at this moment) as well as the following scales:

- State-Trait Anxiety Inventory (STAI) state scale measures current stress levels. STAI responses were rated on a five-point Likert scale ranging from 1 = not at all to 5 = very much so. Pre and post intervention total STAI scores were calculated as the sum of all scale items at each time measure. The STAI Anxiety Inventory scale has good psychometric properties in its original form. In the current study, Cronbach’s alpha values were 0.858 and 0.883 for the pre-and post-intervention scales respectively.
- Positive and Negative Affect Schedule (PANAS) scale consists of 10 items designed to measure mood. Responses were rated on a five-point Likert scale ranging from 1 = very slightly or not at all to 5 = extremely. Total positive and negative affect scores were calculated as the mean of items relevant to each scale. Pre-intervention Cronbach α values were 0.730 and 0.566 for positive and negative affect scales, respectively. Post-intervention Cronbach α values were 0.781 and 0.705 for positive and negative affect scales, respectively.
- Amsterdam Resting-State Questionnaire (ARSQ) aimed to measure state mind wandering. ARSQ scale consisted of 27 items rated on a five-point Likert scale (1 = completely disagree, 5 = completely agree). Total ARSQ scores were calculated as the mean of all items. Cronbach α values were 0.815 and 0.881 for pre and post intervention scales, respectively.

Further questionnaires were used only post-condition:

- Short State Interoceptive Sensibility questionnaire (SSIS). We constructed this questionnaire consisting of 15 items designed to measure the participants’ awareness of their bodies and internal sensations. Following an extensive literature review, no existing questionnaire was found for assessing state interoceptive sensibility with respect to the characteristics relevant to the current study. Items were designated along a five-point Likert scale ranging from 1- not at all to 5- very much so. A total body-awareness score was calculated as the mean of all items. Cronbach alpha was high (α = 0.937).
- The Activity Flow State Scale (AFSS) consists of 26 items designed to assess participants’ flow rating. Items were designated along a five-point Likert scale ranging from 1- strongly disagree to 5- strongly agree. A total flow score was calculated as the mean of all items. Cronbach alpha for the current study was 0.874).
- Embodiment questionnaire: we devised a five item embodiment questionnaire to assess the extent to which the participants perceived a connection between the stimuli presented and their body. This questionnaire served to verify that participants indeed felt a sense of embodiment in the responsive mechanism (Cronbach α = 0.958). Embodiment total score was calculated as the mean of all items. Embodiment score was highly correlated with system use (r = 0.763, p < 0.001). Two individuals from the intervention group reported a lack of embodiment, consequently, these participants were excluded from subsequent analyses.

### Statistical analysis

Following this study’s hypotheses of increased interoceptive sensibility and sense of flow and decreased ARSQ means among system user participants compared with the control group, *T*-tests, repeated measures ANOVA and chi square tests were used for additional comparisons, as relevant.

In order to enhance the transparency of reported statistical results, all statistical analyses were conducted and reported both in classical frequentist and Bayesian methods. Bayesian models were compared to the null model and BF_10_ are reported. BF_10_ values tell us how many times are the data more likely to occure under the model in question compared with the null model. Alternative models yielding a BF_10_ > 1 support the alternative model compared to null model, with higher BF_10_ values providing stronger support for the alternative model.

Post intervention outcomes (interoceptive sensibility, AFSS, ARSQ) predictors were further assessed using three separate hierarchical, linear regression models controlling for background characteristics. These included gender, ADD and meditation experience entered in the first step of each model. The model for ARSQ was adjusted to pre-intervention ARSQ score as well. Pre-intervention measures significant at p < 0.2 in bivariate analyses were entered in the second step, in a stepwise method aimed to build the best model that accounts for the most variance in the outcome variable. Pre-intervention measures included in each final regression model are detailed in the results section. In order to assess the unique contribution of the system-experience (condition) to the prediction of interoceptive sensibility, it was entered separately in step three of each model. The interaction between system-use and each of the following pre-intervention characteristics: gender, ADD, meditation and arousal were assessed for moderating the relationship between system-experience and each of the outcome measures and were entered in the last step. Model properties (residual analysis, change in R^2^, tolerance and VIF values) were assessed to determine model goodness of fit. Statistical analyses were conducted using SPSS vs23.

Bayesian linear regression models were assessed using a uniform prior (which assumes that all models are equally likely before observing the data); each model was compared to the Null model. In addition, we applied a JZS prior (r scale = 0.354) to the regression coefficients. Bayesian statistical analyses were conducted using JASP v0.16.4.

### Supplementary Information

Supplementary Information.

## Data Availability

The data analyzed during the current study is available from the corresponding author on reasonable request.
