# Supplementary material for: Enhancing interoceptive sensibility through exteroceptive–interoceptive sensory substitution
Source: Sci Rep. 2024 Jun 27;14:14855. doi: 10.1038/s41598-024-63231-4 (PMC11211335; doi:10.1038/s41598-024-63231-4)
Supplement: Supplementary file 1 — Supplementary Information. [file 41598_2024_63231_MOESM1_ESM.pdf]

Short State Interoceptive Sensibility Questionnaire

In the following questionnaire you will find several sentences describing the experience you had. Read each item and then rate the extent to which you feel it represents your experience.
(1 - not at all, 5 = very much so)

1. I noticed my breath.
2. I noticed my posture.
3. I noticed the physical sensations.
4. I noticed my heart beat.
5. I was aware of feelings of comfort or discomfort that arose in me.
6. I felt my weight against the chair.
7. I noticed my breathing pattern.
8. I noticed the pace of my breathing.
9. I noticed the expansion of the chest following my breathing.
10. Even when my attention wandered, I brought my awareness back to my body experience.
11. Even when my attention wandered, I brought my awareness back to my breathing.
12. Even when I felt discomfort, I kept my awareness on my breath.
13. My bodily experience was interesting.
14. I actively explored my bodily experience.
15. I felt present and connected to the bodily experience.

בשאלןבא תמצא/י מספר משפטים המתארים את החוויה שעברה עליך.
קרא/י כל פריט ואחר כך סמן/י את המידה בה את/ה חש/ה שהוא מייצג את החוויה שלך.
(1 - בכלל לא, 5 - במידה רבה)

ב לנשימה שלי 2. שמתי לב
לתנוחה שלי
3. שמתי לב לתחושות גופניות שהיו לי
4. שמתי לב לפעימות הלב שלי
5. הייתי מודע לתחושות נוחות או אי נוחות שעלו בי
6. שמתי לב למשקל שלי כנגד הכסא
7. שמתי לב לדפוס הנשימה שלי
8. שמתי לב לקצב הנשימה שלי
9. שמתי לב להתרחבות של בית החזה שהנשימה מייצרת
10. גם כאשר תשומת הלב שלי נדדה, החזרתי את המודעות שלי לחוויה הגופנית
1. גם כאשר תשומת הלב שלי נדדה, החזרתי את המודעות שלי לדפוסי הנשימה שלי
12. גם כאשר הרגשתי חוסר נוחות, שמרתי על מודעות לנשימה שלי
13. החוויה הגופנית שלי הייתה מעניינת
14. חקרתי את החוויה הגופנית שלי באופן פעיל
15. הרגשתי נוכח ומחובר לחוויה הגופנית
